# Housing at 27 °C, a Near-Thermoneutral Temperature, Exacerbates Metabolic Dysfunction-Associated Steatohepatitis in C57BL/6J Mice

**DOI:** 10.3390/ijms27177621

**Published:** 2026-08-25

**Authors:** Lei Hao, Md Shahjalal Hossain Khan, Yujiao Zu, Shu Wang

**Affiliations:** 1 Department of Nutritional Sciences, Texas Tech University, Lubbock, TX 79409, USA; 2 Department of Allied Health, Indiana University of Pennsylvania, Indiana, PA 15705, USA; 3 College of Health Solutions, Arizona State University, Phoenix, AZ 85004, USA

**Keywords:** brown adipose tissue, fibrosis, hepatic steatosis, inflammation, metabolic dysfunction-associated steatohepatitis, obesity, thermogenesis

## Abstract

Metabolic dysfunction-associated steatohepatitis (MASH), a progressive form of metabolic dysfunction-associated steatotic liver disease, affects approximately 3–5% of the global population and lacks effective therapies. This study examined the impact of near-thermoneutral housing on MASH progression in mice fed a fast-food diet (FFD) enriched in fat, sucrose, and cholesterol. Male C57BL/6J mice were housed under standard (22 °C) or near-thermoneutral (27 °C) conditions and fed chow or FFD for 20 weeks. Body weight, composition, liver pathology, hepatic gene expression, plasma lipids, and adipose thermogenic and inflammatory markers were assessed. FFD increased body weight, fat mass, and lean mass at both temperatures. Near-thermoneutral housing markedly exacerbated MASH in FFD-fed mice, with increased steatosis, fibrosis, inflammation, and elevated liver enzymes. Hepatic lipogenic and fibrosis-related gene expression was upregulated, alongside higher plasma total cholesterol and very-low-density lipoprotein levels. Near-thermoneutral housing also impaired brown adipose tissue thermogenesis, reducing uncoupling protein 1 and other thermogenic genes, and modestly altered inguinal white adipose tissue inflammation and thermogenic signaling. These findings demonstrate that near-thermoneutral housing accelerates MASH progression, accompanied by increased hepatic lipid accumulation, fibrosis, and inflammation and reduced adipose thermogenic signatures. These findings identify environmental temperature as a critical modifier of MASH pathophysiology and suggest that enhancing adipose thermogenesis may offer therapeutic potential.

## 1. Introduction

The liver is one of the key metabolic organs in the human body. After absorption, most nutrients are transported to the liver for storage, metabolism, and utilization. Consequently, the liver is highly susceptible to external stimuli, such as nutrient excess, alcohol toxicity, and other metabolic stressors [1]. Among liver diseases, metabolic dysfunction-associated steatotic liver disease (MASLD), formerly called nonalcoholic fatty liver disease, is the most common chronic liver condition [2,3], characterized by lipid accumulation in hepatocytes. MASLD can range from simple steatosis, which is generally reversible with lifestyle modifications, to more severe forms such as metabolic dysfunction-associated steatohepatitis (MASH) and cirrhosis [4]. While lifestyle changes may help manage MASH, there is limited evidence on whether they can fully reverse the condition.

MASLD affects approximately 30% of the global population [5], with a particularly high prevalence among individuals with obesity [6]. However, many affected individuals remain unaware of their condition. Among those with MASLD, about 10–30% may progress to MASH [7], and approximately 3–5% may develop cirrhosis, which can further lead to hepatocellular carcinoma in some cases [8]. The current strategy for managing MASH relies primarily on lifestyle modifications [9], complemented by pharmacological interventions for associated conditions [9,10]. For instance, antidiabetic medications like liraglutide have been proposed as a treatment option for MASLD patients with comorbid type 2 diabetes [11].

Drug development for MASH primarily targets multiple pathways, including lipogenesis, glucose metabolism, bile acid metabolism, inflammation, and fibrosis [6,12,13]. Currently, Rezdiffra (resmetirom) and Wegovy (semaglutide) are the only medications approved by the Food and Drug Administration for the treatment of MASH [14,15]. Resmetirom achieves a histological response in fewer than 30% of patients [16], whereas semaglutide significantly improved histologic outcomes in MASH, with resolution of steatohepatitis without worsening of fibrosis achieved in 62.9% of patients compared with 34.3% in the placebo group and fibrosis improvement without worsening of steatohepatitis observed in 36.8% versus 22.4%, respectively [17], highlighting heterogeneity in treatment response and the ongoing need for novel preventive and therapeutic strategies. 

Over the past decade, brown adipose tissue (BAT) has garnered significant interest in the field of nutrition and metabolism due to its unique role in energy expenditure through thermogenesis [18,19]. Unlike white adipose tissue (WAT), which primarily functions as an energy reservoir, BAT dissipates chemical energy as heat through non-shivering thermogenesis and can contribute to whole-body energy expenditure when activated. Although the precise contribution of BAT to energy expenditure in humans remains under debate, metabolically active BAT has the potential to make a meaningful contribution to daily energy expenditure [20,21].

Beyond its thermogenic properties, BAT plays a crucial role in hepatic lipid metabolism. The liver and adipose tissue work in concert to regulate fatty acid flux from dietary fat intake, de novo lipogenesis, and lipolysis [22]. Excessive lipolysis can overwhelm the liver, contributing to the development of MASLD. However, BAT can utilize surplus fatty acids, alleviating the metabolic burden on the liver. Additionally, BAT secretes neuregulin 4 (Nrg4), an endocrine factor that has been shown to protect against diet-induced hepatic steatosis by promoting fatty acid oxidation and ketogenesis while reducing hepatic lipogenesis [23,24].

Additionally, beige adipocytes are inducible thermogenic cells that develop within WAT in response to stimuli such as cold exposure, exercise, and β-adrenergic activation [25]. Browning of WAT increases energy expenditure and fatty acid oxidation through both UCP1-dependent and UCP1-independent thermogenic pathways, thereby improving systemic glucose and lipid metabolism and potentially reducing hepatic lipid accumulation [26,27].

Animal and human studies further support the role of BAT in regulating hepatic lipid metabolism and mitigating MASLD risk. Research has demonstrated that BAT implantation prevents high-fat diet-induced hepatic steatosis in mice [28], while BAT transplantation reverses hepatic steatosis in leptin-deficient ob/ob mice [29]. Moreover, studies indicate that the loss of beige adipocytes in PR domain-containing 16 knockout mice leads to hepatic insulin resistance and steatosis [30]. Retrospective human studies have also revealed a significant inverse correlation between the presence of BAT and MASLD prevalence in adults [31,32]. Given these findings, targeting extrahepatic tissues by enhancing the activity of BAT has emerged as a promising therapeutic strategy for managing MASLD and its advanced stage, MASH. 

Building on our previous findings that housing at 27 °C, a near-thermoneutral temperature, exacerbates liver steatosis [33], our current study aims to investigate its role in the progression of MASH, the more advanced stage of MASLD. Here, the term “near-thermoneutral” is used descriptively to refer to a housing temperature intermediate between conventional laboratory housing (22 °C) and the commonly used thermoneutral temperature range (29–30 °C) in mice. Therefore, 27 °C should not be interpreted as fully thermoneutral conditions. Understanding the impact of near-thermoneutral temperature on MASH development will provide further insight into the metabolic mechanisms underlying liver disease and may inform new strategies for prevention and treatment.

## 2. Results

### 2.1. Body Weight Was Altered by Diet and Housing Temperature

**Figure 1:**
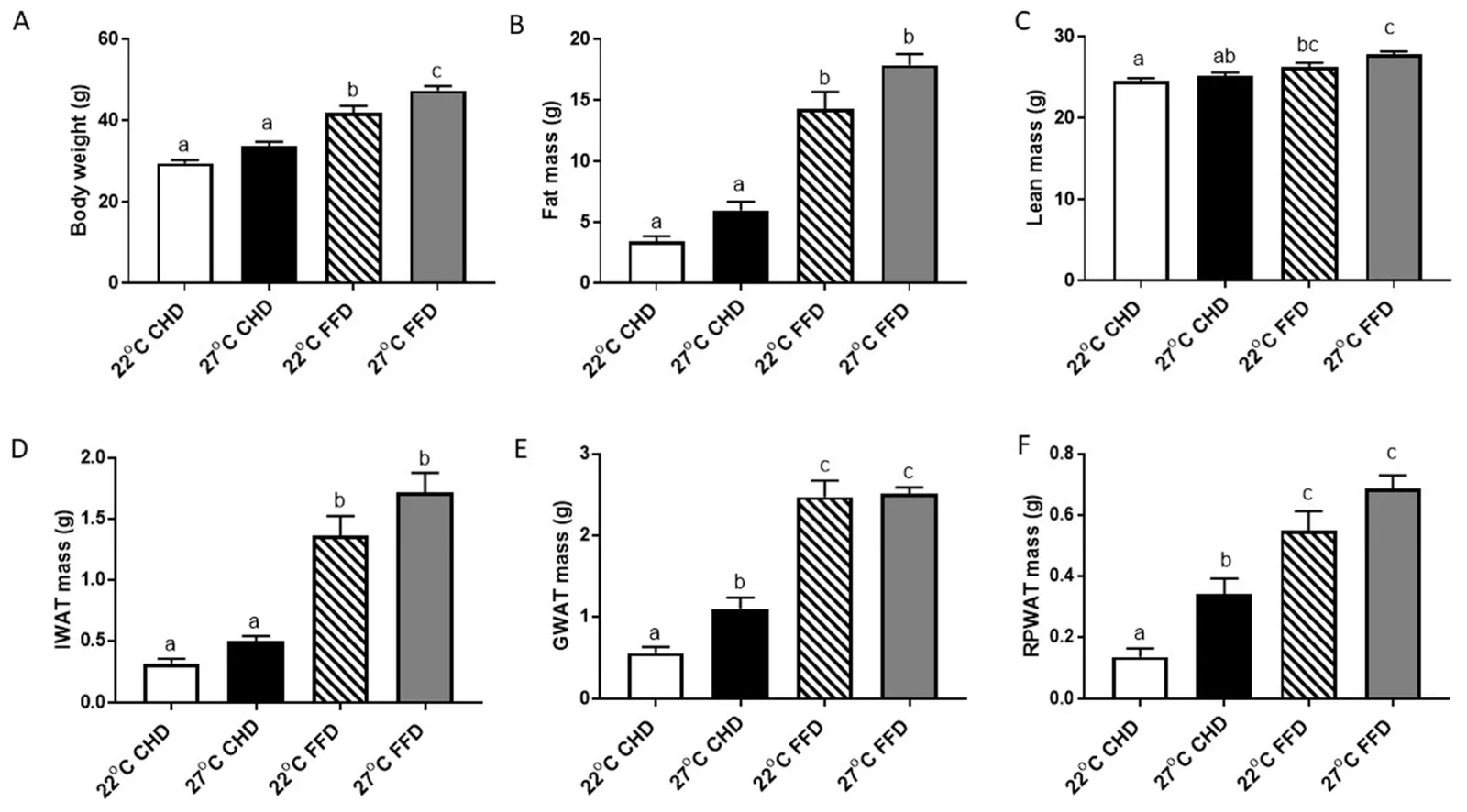
Effects of diet and housing temperature on body weight, body composition, and adipose tissue mass: (**A**) final body weight, (**B**) fat mass, (**C**) lean mass, (**D**) inguinal white adipose tissue (IWAT) mass, (**E**) gonadal white adipose tissue (GWAT) mass, and (**F**) retroperitoneal white adipose tissue (RPWAT) mass. Data are presented as means ± SEM (*n* = 8 per group). Statistical analysis was performed using two-way ANOVA, followed by Tukey’s multiple-comparison test. Different letters indicate statistically significant differences (*p* < 0.05).

After 20 weeks of feeding, mice on the FFD diet were significantly heavier than those on the control chow diet (CHD) diet, regardless of housing temperature (Figure 1A). Housing at 27 °C significantly increased the final body weight of FFD-fed mice by approximately 13% (47.30 vs. 41.89 g) compared with 22 °C (*p* = 0.0154) but not in CHD-fed mice (Figure 1A) (*p* = 0.2731). Body composition analysis confirmed that FFD-fed mice had greater fat mass than their CHD-fed counterparts, regardless of housing temperature (Figure 1B). Additionally, FFD-fed mice at 27 °C exhibited a numerical increase in fat mass compared with those at 22 °C (25% increase, *p* = 0.0574); however, this difference did not reach statistical significance (Figure 1B).

Body composition analysis also demonstrated that FFD feeding resulted in significantly higher lean mass across temperatures (Figure 1C). Fat depots, including inguinal WAT (IWAT), gonadal WAT (GWAT), and retroperitoneal WAT (RPWAT), were larger in FFD-fed mice compared to CHD-fed mice (Figure 1D–F). Temperature did not alter fat depot mass in FFD-fed mice; however, housing at 27 °C increased GWAT mass by 98% (*p* = 0.0397) and RPWAT mass by 152% (*p* = 0.0280) in CHD-fed mice compared with 22 °C (Figure 1E,F).

Calorie intake was monitored weekly throughout the 20-week intervention period to determine whether differences in energy intake contributed to the observed metabolic phenotype. Weekly calorie intake was lower in mice housed at 27 °C compared with those housed at 22 °C in both dietary groups. Specifically, calorie intake was 87.4 kcal/week in chow-fed mice at 22 °C, 67.0 kcal/week in chow-fed mice at 27 °C, 99.3 kcal/week in FFD-fed mice at 22 °C, and 80.7 kcal/week in FFD-fed mice at 27 °C (Supplementary Figure S1).

### 2.2. Housing at 27 °C Improved Glucose Tolerance but Did Not Alter Insulin Sensitivity

**Figure 2:**
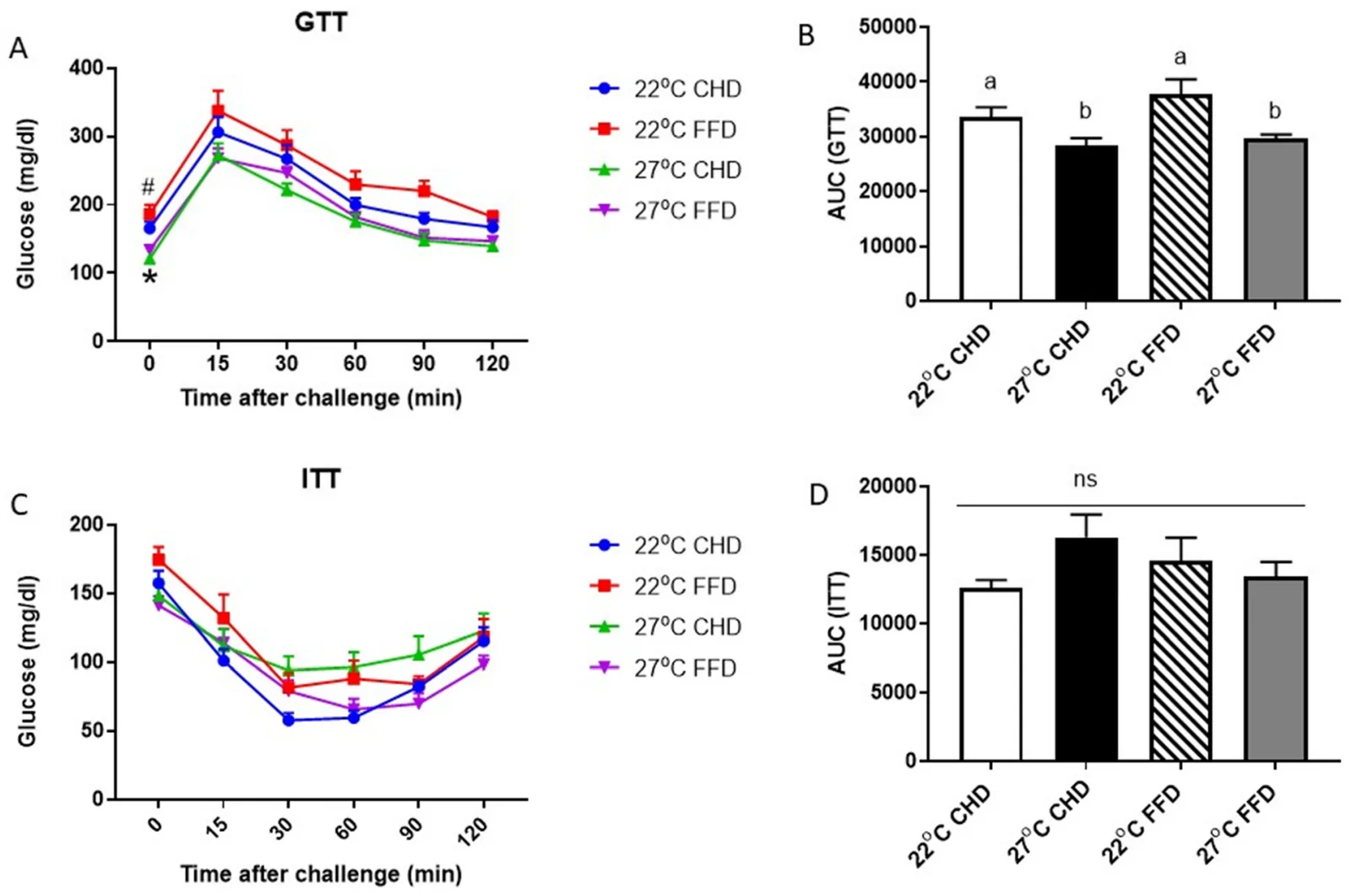
Effects of housing temperature and diet on glucose tolerance and insulin sensitivity: (**A**) Glucose tolerance test (GTT) curves following glucose administration; * indicates a significant difference between the 27 °C CHD and 22 °C CHD groups, and # indicates a significant difference between the 27 °C FFD and 22 °C FFD groups. (**B**) Quantification of glucose tolerance by calculating the area under the curve (AUC) of the GTT. Different letters indicate significant differences among groups. (**C**) Insulin tolerance test (ITT) curves following insulin administration. (**D**) Quantification of insulin sensitivity by calculating the AUC value of the ITT. Data are presented as mean ± SEM. GTT and ITT AUC values were analyzed by ordinary two-way ANOVA with housing temperature (22 °C vs. 27 °C) and diet (CHD vs. FFD) as factors, followed by Tukey’s multiple-comparison tests. Statistical significance was defined as *p* < 0.05. “ns” indicates no significant difference.

Glucose tolerance and insulin sensitivity were assessed approximately one week before the completion of the 20-week dietary and housing intervention. Fasting blood glucose concentrations were analyzed by two-way ANOVA with housing temperature and diet as factors. Housing temperature had a significant effect on fasting glucose levels (*p* < 0.0001), with mice housed at 27 °C exhibiting lower fasting glucose concentrations compared with those housed at 22 °C (127.6 vs. 175.8 mg/dL). Neither diet (*p* = 0.101) nor the housing temperature × diet interaction (*p* = 0.697) significantly affected fasting glucose levels (Figure 2A).

Glucose tolerance was assessed by calculating the area under the curve (AUC) of the glucose tolerance test (GTT). Mean GTT AUC values were 33,555 ± 1797 for the 22 °C CHD group, 37,768 ± 2704 for the 22 °C FFD group, 28,436 ± 1277 for the 27 °C CHD group, and 29,666 ± 671 for the 27 °C FFD group (mean ± SEM). Two-way ANOVA revealed a significant main effect of housing temperature on GTT AUC (*p* = 0.0012), with mice housed at 27 °C exhibiting significantly lower GTT AUC values than those housed at 22 °C. Neither diet (*p* = 0.149) nor the housing temperature × diet interaction (*p* = 0.423) was significant, indicating that the effect of housing temperature on glucose tolerance was independent of dietary intervention (Figure 2B).

Insulin sensitivity was evaluated by calculating the AUC of the insulin tolerance test (ITT). Two-way ANOVA showed no significant effects of housing temperature (*p* = 0.347) or diet (*p* = 0.756) on ITT AUC. Although the housing temperature × diet interaction did not reach statistical significance (*p* = 0.080), a trend toward an interaction was observed (Figure 2C,D).

### 2.3. Housing at 27 °C Worsened MASH in FFD-Fed Mice

**Figure 3:**
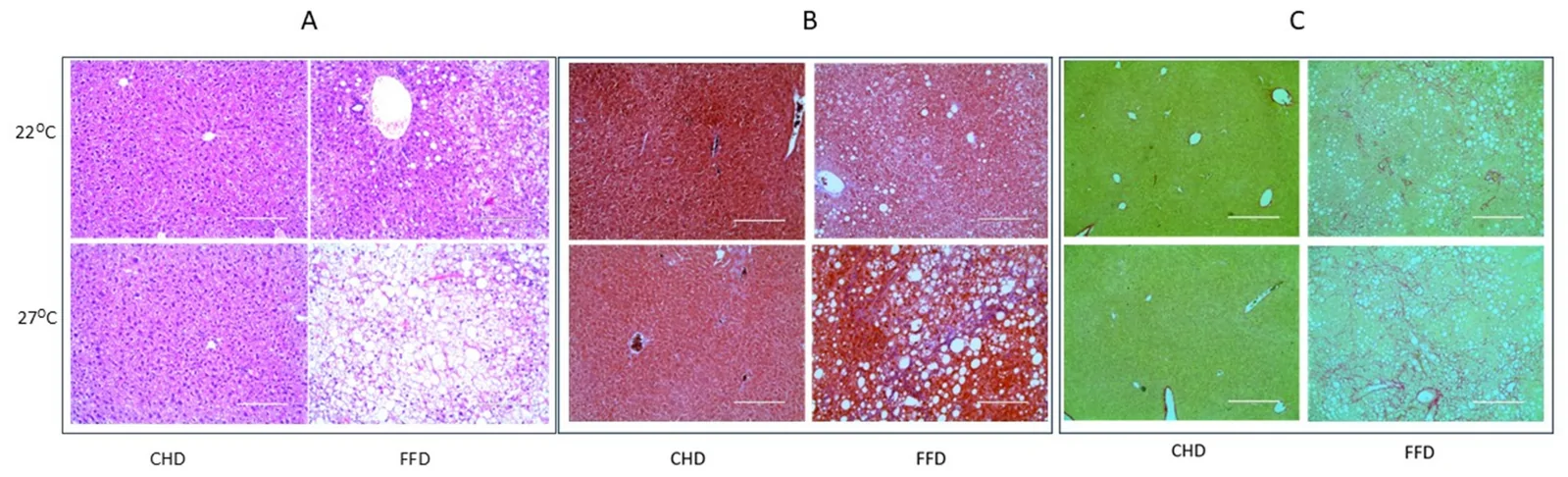
Histological evidence of MASH worsening in FFD-fed mice housed at 27 °C: (**A**) hematoxylin and eosin (H&E) staining of the liver (scale bar: 200 μm); (**B**) trichrome staining of the liver (scale bar: 200 μm); (**C**) Picrosirius Red staining of the liver (scale bar: 200 μm).

Histological assessment of the liver, including H&E staining, trichrome staining, and Picrosirius Red staining, revealed more pronounced lipid accumulation and collagen deposition in FFD-fed mice housed at 27 °C compared to those housed at 22 °C (Figure 3A–C). H&E staining showed no visible lipid accumulation in CHD-fed mice, regardless of housing temperature (Figure 3A). Consistently, trichrome and Picrosirius Red staining confirmed the absence of collagen formation in CHD-fed mice across both temperatures (Figure 3B,C). In contrast, FFD-fed mice exhibited substantial lipid accumulation and collagen deposition, as evidenced by all three staining methods (Figure 3A–C). Notably, these pathological changes were significantly exacerbated in FFD-fed mice housed at 27 °C compared with those housed at 22 °C (Figure 3A–C).

**Figure 4:**
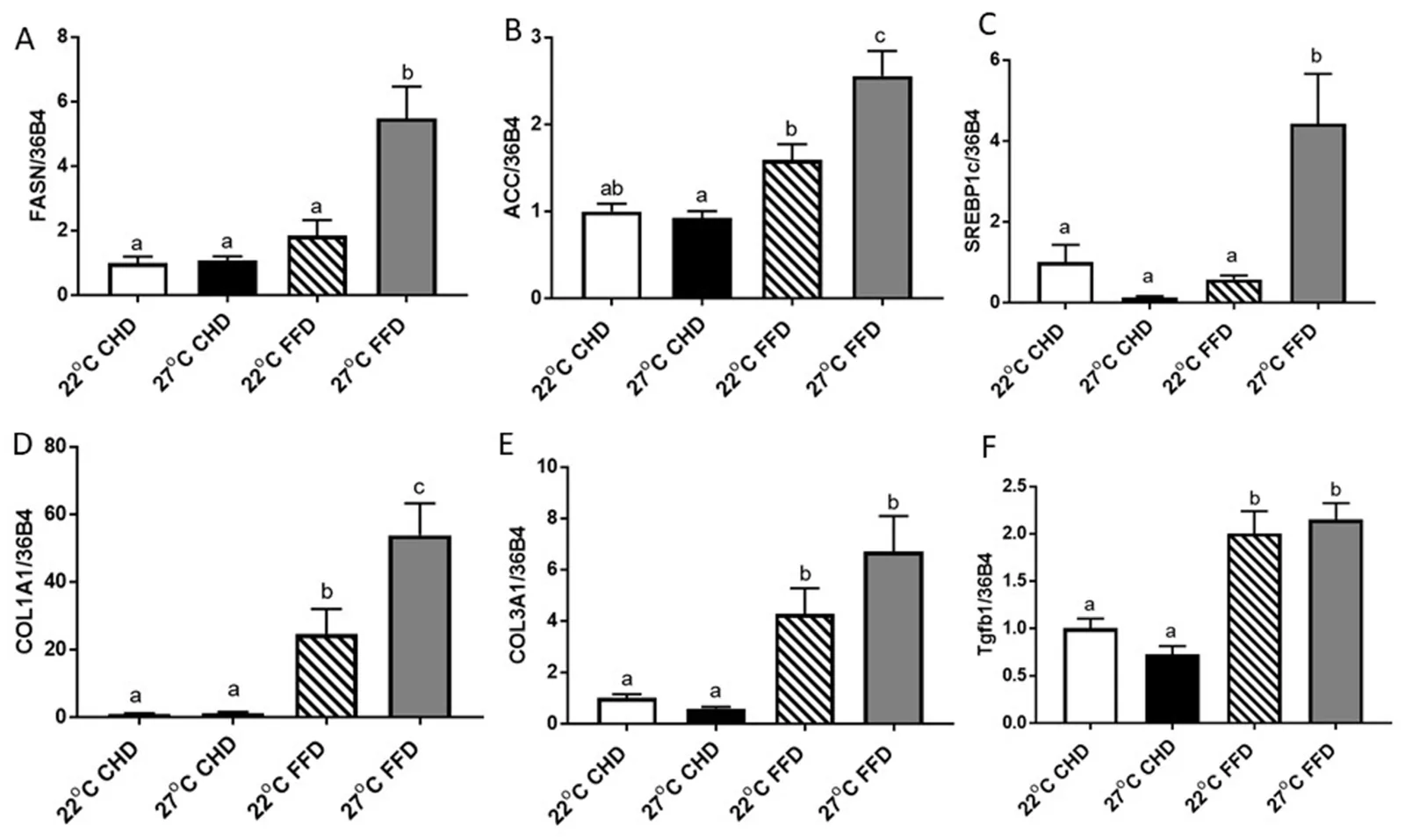
Housing at 27 °C worsened MASH in FFD-fed mice. Gene expression analysis of (**A**) *Fasn*, (**B**) *Acc*, (**C**) *Srebp1c*, (**D**) *Col1a1*, (**E**) *Col3a1*, and (**F**) *Tgfb1*. Data are presented as means ± SEM (*n* = 8 per group). Statistical analysis was performed using two-way ANOVA, followed by Tukey’s multiple-comparison test. Different letters indicate statistically significant differences (*p* < 0.05).

Real-time (RT) PCR analysis further supported these findings, showing that at 27 °C, FFD feeding significantly upregulated lipogenic gene expression, with fatty acid synthase (*Fasn*) increased by 196%, acetyl-CoA carboxylase 1 (*Acc1*) by 61%, and sterol regulatory element binding protein 1c (*Srebp1c*) by 665%, compared with mice maintained at 22 °C (Figure 4A–C). Additionally, collagen formation-related genes were significantly upregulated in FFD-fed mice compared with CHD-fed mice at 22 °C, including collagen type I alpha 1 (*Col1a1*), which increased by 2361%; collagen type III alpha 1 chain (*Col3a1*), by 326%; and transforming growth factor beta 1 (*Tgfb1*), by 100%. Furthermore, *Col1a1* expression was increased by 120% in FFD-fed mice at 27 °C compared with those at 22 °C (Figure 4D–F).

**Figure 5:**
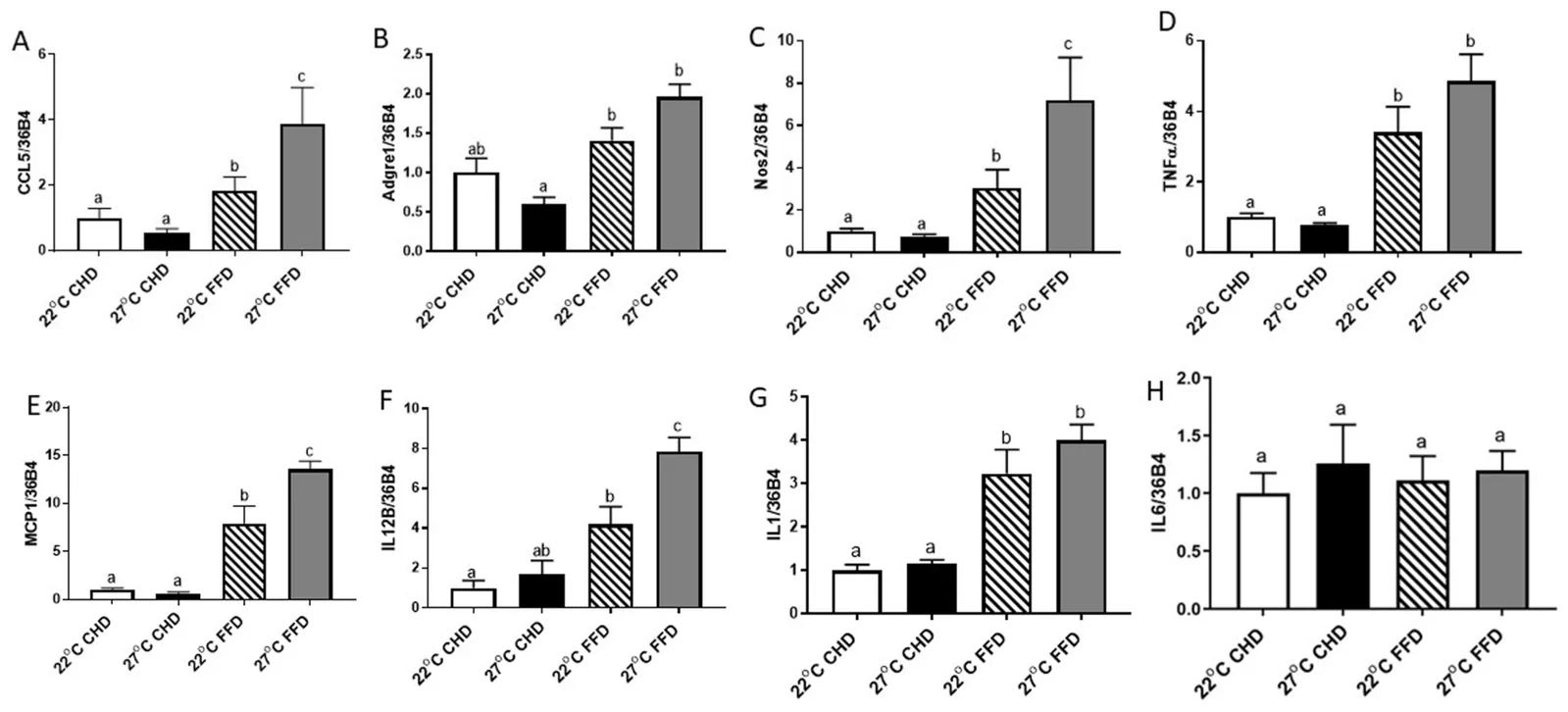
Housing at 27 °C and FFD feeding increase inflammatory gene expression in the liver. Gene expression analysis of (**A**) *Ccl5*, (**B**) *Adgre1*, (**C**) *Nos2*, (**D**) *Tnfα*, (**E**) *Mcp1*, (**F**) *Il12b*, (**G**) *Il1*, and (**H**) *Il6*. Data are presented as means ± SEM (*n* = 8 per group). Statistical analysis was performed using two-way ANOVA, followed by Tukey’s multiple-comparison test. Different letters indicate statistically significant differences (*p* < 0.05).

RT-PCR also revealed significant upregulation of inflammation markers in FFD-fed mice compared with CHD-fed mice at 22 °C, including chemokine (C-C motif) ligand 5 (*Ccl5*) by 82%, adhesion G protein-coupled receptor E1 (*Adgre1*) by 41%, nitric oxide synthase 2 (*Nos2*) by 203%, tumor necrosis factor alpha-like (*Tnfα*) by 241%, chemokine (C-C motif) ligand 2 (*Ccl2* or *Mcp1*) by 693%, interleukin-12 subunit beta (*Il12b*) by 318%, and interleukin-1 (*Il1*) by 223% (Figure 5A–G). Additionally, among these inflammatory markers, *Ccl5*, *Nos2*, *Mcp1*, and *Il12b* were significantly increased by 110%, 140%, 71%, and 88%, respectively, in FFD-fed mice housed at 27 °C compared with those at 22 °C (Figure 5A,C,E,F). *Il6* expression remained unchanged across both diet and temperature conditions (Figure 5H).

**Figure 6:**
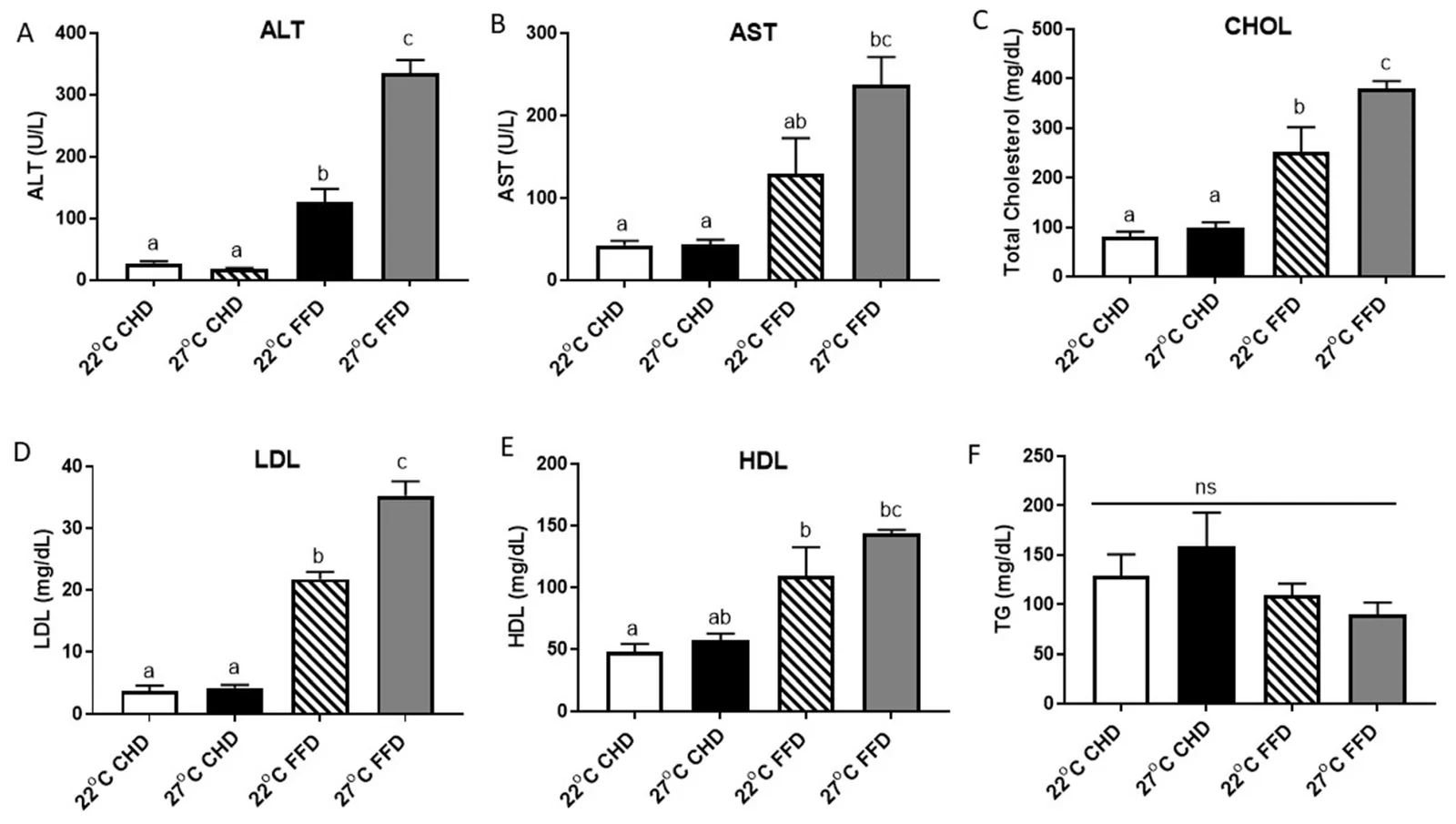
Housing at 27 °C and FFD feeding impair liver function and alter the lipid profile: (**A**) alanine aminotransferase (ALT), (**B**) aspartate aminotransferase (AST), (**C**) total cholesterol, (**D**) low-density lipoprotein (LDL), (**E**) high-density lipoprotein (HDL), and (**F**) triglycerides (TGs). Data are presented as means ± SEM (*n* = 4 per group). Statistical analysis was performed using two-way ANOVA, followed by Tukey’s multiple-comparison test. Different letters indicate statistically significant differences (*p* < 0.05). ns, not statistically significant; horizontal lines indicate the groups being compared statistically.

Liver function analysis revealed that FFD feeding, compared with CHD feeding, significantly elevated plasma levels of alanine aminotransferase (ALT) and aspartate aminotransferase (AST) by 364% and 210%, respectively, in mice housed at 22 °C. Furthermore, ALT levels were significantly increased by 164% in FFD-fed mice at 27 °C compared with those at 22 °C (Figure 6A). Similarly, AST levels were elevated by 86% in FFD-fed mice housed at 27 °C; however, this increase did not reach statistical significance due to variation (Figure 6B).

Plasma lipid analysis demonstrated that FFD feeding significantly increased total cholesterol by 209% (Figure 6C), low-density lipoproteins (LDL) by 480% (Figure 4D), and high-density lipoproteins (HDL) by 129% in mice housed at 22 °C (Figure 6E). Triglyceride (TG) levels remained unaffected by diet (Figure 6F). However, housing at 27 °C significantly elevated total cholesterol levels by 50% in FFD-fed mice compared with those maintained at 22 °C.

Taken together, both histological and RT-PCR data demonstrate that 27 °C exacerbates MASH in FFD-fed mice.

### 2.4. Housing at 27 °C Suppressed Thermogenesis Markers in BAT

**Figure 7:**
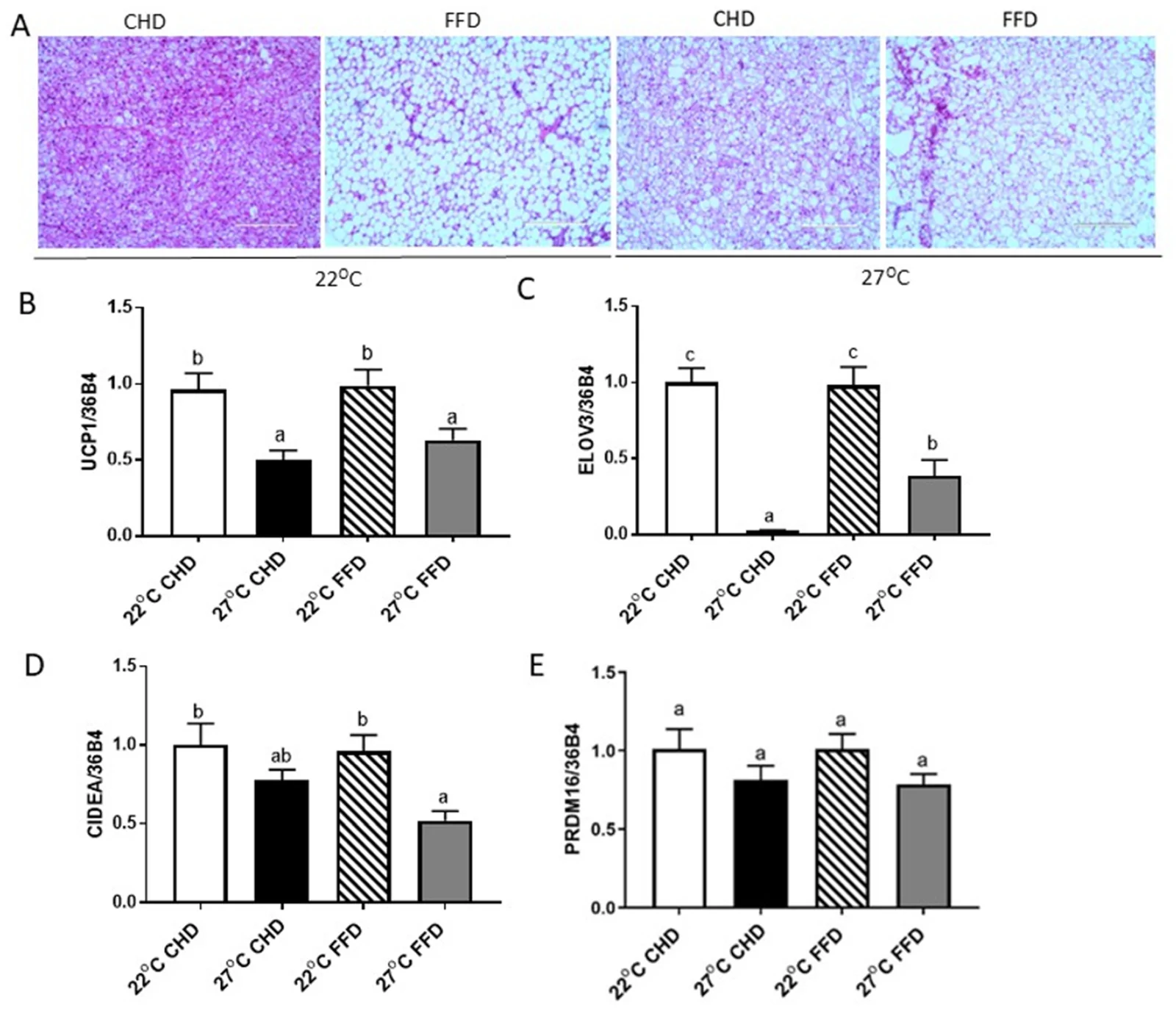
Housing at 27 °C inhibits expression of thermogenesis markers in BAT: (**A**) hematoxylin and eosin (H&E) staining of BAT (scale bar: 200 μm), (**B**) *Ucp1* mRNA expression, (**C**) *Elov3* mRNA expression, (**D**) Cidea mRNA expression, and (**E**) *Prdm16* mRNA expression. Data are presented as means ± SEM (*n* = 4 per group for histology, *n* = 8 per group for gene expression). Statistical analysis was performed using two-way ANOVA, followed by Tukey’s multiple-comparison test. Different letters indicate statistically significant differences (*p* < 0.05).

H&E staining of BAT revealed distinct morphological differences across diet and temperature conditions. CHD-fed mice at 22 °C exhibited normal BAT morphology, characterized by abundant small multilocular lipid droplets, indicative of active thermogenesis. In contrast, CHD-fed mice at 27 °C showed a mix of small multilocular and large unilocular lipid droplets, suggesting partial whitening of BAT. FFD-fed mice exhibited more pronounced BAT whitening across both temperatures, with predominantly large unilocular lipid droplets and minimal multilocular droplets, indicating impaired thermogenic capacity (Figure 7A).

RT-PCR analysis revealed that 27 °C significantly downregulated key thermogenesis markers in BAT of CHD-fed mice, including *Ucp1* by 48%, ELOVL fatty acid elongase 3 (*Elov3*) by 98%, and cell death-inducing DNA fragmentation factor alpha-like effector A (*Cidea*) by 22% (Figure 7B–D). Similarly, in FFD mice, 27 °C significantly reduced BAT expression of *Ucp1* by 36%, *Elov3* by 61%, and *Cidea* by 46% (Figure 7B–D). However, PR domain-containing 16 (*Prdm16*) expression remained unchanged across all groups (Figure 7E).

### 2.5. FFD Feeding Increased Adiposity and Inflammation, While 27 °C Slightly Inhibited Thermogenesis Markers in IWAT

**Figure 8:**
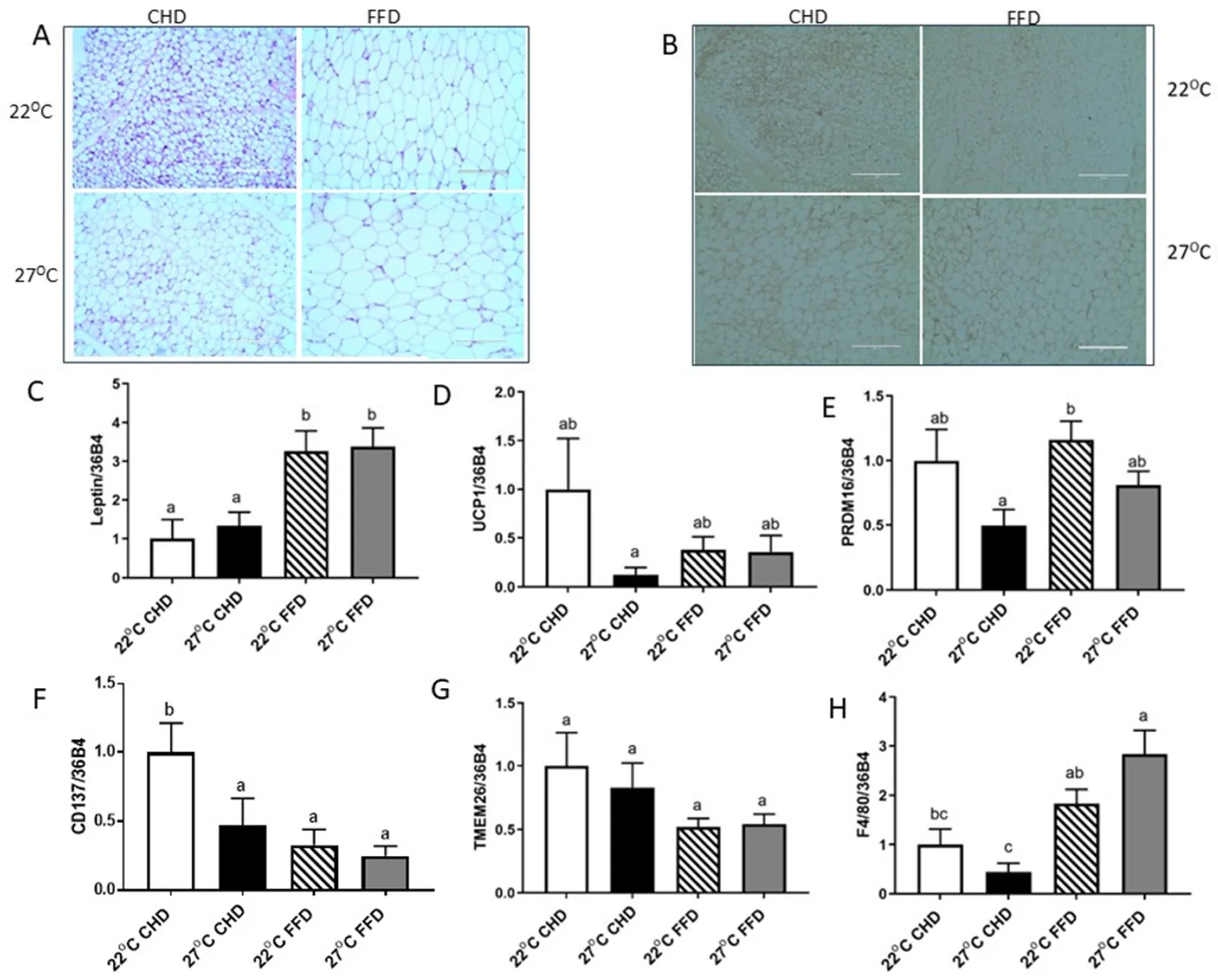
FFD feeding increases adiposity and inflammation, while 27 °C slightly inhibits thermogenesis markers in IWAT: (**A**) hematoxylin and eosin (H&E) staining of IWAT (scale bar: 200 μm), (**B**) leptin mRNA expression (scale bar: 200 μm), (**C**) immunohistochemical staining of UCP1 in IWAT, (**D**) *Ucp1* mRNA expression, (**E**) *Prdm16* mRNA expression, (**F**) *Cd137* mRNA expression, (**G**) *Tmem26* mRNA expression, and (**H**) *F4/80* mRNA expression. Data are presented as means ± SEM (*n* = 8 per group for gene expression). Statistical analysis was performed using two-way ANOVA, followed by Tukey’s multiple-comparison test. Different letters indicate statistically significant differences (*p* < 0.05).

H&E staining of IWAT revealed that FFD feeding significantly increased adipocyte size compared to CHD-fed mice under both 22 °C and 27 °C conditions (Figure 8A).

Immunohistochemical staining of UCP1 in IWAT demonstrated the highest UCP1 protein abundance in CHD-fed mice at 22 °C among all groups (Figure 8B).

RT-PCR analysis showed that FFD feeding increased leptin gene expression, consistent with the observed increase in adipocyte size under both 22 °C and 27 °C conditions (Figure 8C). RT-PCR analysis showed a trend toward decreased *Ucp1* gene expression in CHD-fed mice at 27 °C compared to 22 °C, although this difference did not reach statistical significance (Figure 8D).

Additionally, 27 °C slightly suppressed *Prdm16* and tumor necrosis factor receptor superfamily member 9 (*Tnfrsf9* or *Cd137*) expression in CHD-fed mice, but this effect was not statistically significant (Figure 8E,F). Transmembrane protein 26 (*Tmem26*) expression remained unchanged across all 4 groups (Figure 8G). Furthermore, RT-PCR analysis revealed that FFD feeding significantly increased adhesion G protein-coupled receptor E1 (*Adgre1* or *F4/80*) gene expression by 545% compared to CHD feeding in mice housed at 27 °C but not at 22 °C (Figure 8H), suggesting enhanced macrophage infiltration in IWAT.

## 3. Discussion

Our study demonstrates that 27 °C, a near-thermoneutral temperature, exacerbates the progression of MASH in FFD-fed mice, as evidenced by increased hepatic lipid accumulation, collagen deposition, inflammation, liver damage, and altered adipose tissue metabolism. While previous studies have shown that thermoneutrality promotes obesity and metabolic dysfunction [34,35], our findings extend this knowledge by providing compelling evidence that higher ambient temperatures worsen MASH pathology. Importantly, 27 °C alone did not induce MASH in mice fed CHD, indicating that diet is a critical factor in the development of MASH. While other factors, such as genetics, obesity, and insulin resistance, may also contribute to MASH pathogenesis [36], our study highlights the central role of dietary composition.

The FFD used in our study, which mimics the Western diet, contained 55% fructose and 45% glucose—a composition commonly found in sugary beverages like soda. Remarkably, this FFD successfully induced a MASH model within 20 weeks, characterized by lipid accumulation, inflammation, hepatocellular damage, and collagen fiber formation in the liver. Epidemiological studies have consistently linked excessive carbohydrate intake, particularly fructose, to MASLD [37,38]. Excessive intake of added fructose, particularly from sugar-sweetened beverages and processed foods, has been associated with metabolic disorders such as obesity, type 2 diabetes, and cardiovascular disease [39]. In contrast, fructose consumed as part of whole fruits has generally not been associated with these adverse outcomes and may even be protective, likely because whole fruits provide dietary fiber and other bioactive compounds that support a wide range of health benefits [40,41]. However, our study cannot attribute the development of MASH solely to excessive fructose intake, as the MASH model reflects the combined effects of the entire dietary pattern, including high-fat and high-fructose corn syrup, rather than any single nutrient or food item.

Interestingly, we found that the weights of GWAT and RPWAT in CHD-fed mice were markedly increased, although housing at 27 °C did not significantly affect total fat mass in FFD-fed mice. This finding suggests that reducing cold stress, even in the absence of dietary challenge, may influence regional adipose tissue distribution. In contrast, the absence of a similar effect in FFD-fed mice suggests that diet-induced metabolic alterations may have masked or outweighed the more subtle effects of reduced cold stress on visceral fat deposition. Because energy expenditure was not assessed in this study, we cannot determine whether these changes were associated with altered thermogenic energy expenditure or other mechanisms regulating regional lipid storage. Therefore, the mechanisms underlying this depot-specific adipose tissue redistribution remain to be elucidated.

We next examined whether housing temperature and diet affected glucose metabolism by assessing fasting glucose levels, glucose tolerance, and insulin sensitivity. The present findings are consistent with our previous observation that 27 °C improves glucose regulation compared with conventional housing conditions [33]. In this study, housing mice at 27 °C significantly reduced fasting blood glucose concentrations regardless of dietary intervention, indicating that ambient temperature has a strong influence on basal glucose homeostasis independent of dietary composition. In contrast, glucose tolerance assessed by GTT AUC was affected by both housing temperature and dietary intervention, suggesting that housing temperature and diet may independently contribute to the regulation of postprandial glucose handling. Although the underlying mechanisms were not directly investigated, the improved glucose control observed under near-thermoneutral conditions may involve alterations in energy metabolism, substrate utilization, and hepatic glucose regulation. Interestingly, improved glucose tolerance was not accompanied by significant changes in insulin sensitivity based on ITT results. This suggests that the enhanced glucose disposal observed in near-thermoneutral mice may not be primarily mediated by improved peripheral insulin responsiveness but may involve other regulatory processes, including changes in hepatic glucose production, pancreatic insulin secretion, or insulin-independent glucose uptake. Future studies incorporating glucose clamp techniques, insulin secretion assessments, and tissue-specific metabolic analyses are needed to further elucidate how ambient temperature modulates glucose homeostasis.

Histological analysis revealed that FFD-fed mice housed at 27 °C exhibited more severe hepatic steatosis and fibrosis compared to those housed at standard room temperature (22 °C). Lipid accumulation was markedly increased in FFD-fed mice under near-thermoneutral temperature, as indicated by H&E staining, while collagen deposition was significantly enhanced, as shown by trichrome and Picrosirius Red staining. These findings align with previous research indicating that higher environmental temperatures impair metabolic adaptation and promote lipid storage in the liver [33,42,43]. Gene expression analysis further supported these observations, showing significant upregulation of lipogenic genes (*Fasn*, *Acc1*, and *Srebp1c*) in FFD-fed mice housed at 27 °C compared to those at 22 °C. This suggests that near-thermoneutral temperature enhances hepatic de novo lipogenesis, thereby contributing to increased lipid accumulation. Additionally, the upregulation of fibrogenic genes (*Col1a1*, *Col3a1*, and *Tgfb1*) in FFD-fed mice at 27 °C further supports the notion that near-thermoneutral temperature accelerates fibrosis progression in MASH. Notably, *Col1a1* expression was significantly higher in FFD-fed mice at 27 °C compared to 22 °C, reinforcing the role of near-thermoneutral temperature in exacerbating hepatic fibrogenesis.

Inflammation is a key driver of MASH progression, and our findings indicate that near-thermoneutral temperature intensifies hepatic inflammation in FFD-fed mice. RT-PCR analysis revealed significant upregulation of inflammation markers (*Ccl5*, *Adgre1*, *Nos2*, *Tnfα*, *Mcp1*, *Il12b*, and *Il1*) in FFD-fed mice compared to CHD-fed mice, with *Ccl5*, *Nos2*, *Mcp1*, and *Il12b* showing even higher expression at 27 °C. These results suggest that near-thermoneutral temperature amplifies pro-inflammatory signaling, potentially accelerating MASH pathogenesis. This finding aligns with previous reports showing that higher ambient temperatures impair immune cell function and exacerbate liver inflammation [42].

BAT plays a crucial role in non-shivering thermogenesis, driven primarily by UCP1-mediated mitochondrial uncoupling. Our findings show that 27 °C significantly downregulated molecular markers associated with BAT thermogenesis, as evidenced by reduced *Ucp1* gene expression and impaired morphological characteristics. In CHD-fed mice at 22 °C, BAT exhibited abundant small multilocular lipid droplets, indicative of active thermogenesis. However, at 27 °C, brown adipocytes contained a mix of multilocular and unilocular lipid droplets, suggesting a shift toward BAT whitening, a process associated with decreased energy expenditure. In FFD-fed mice, BAT whitening was even more pronounced, with predominantly large unilocular lipid droplets and minimal multilocular structures, regardless of temperature. Gene expression analysis further supported these histological findings, as RT-PCR showed significant downregulation of thermogenesis-related genes (*Ucp1*, *Elov3*, and *Cidea*) at 27 °C, irrespective of diet. However, *Prdm16* expression remained unchanged, suggesting that a near-thermoneutral temperature primarily affects BAT function rather than its differentiation status. These results are consistent with previous studies demonstrating that higher ambient temperatures suppress BAT thermogenic activity, reduce energy expenditure, and promote metabolic dysfunction [33,44]. Given the critical role of BAT in lipid utilization and systemic energy homeostasis, thermoneutrality-induced BAT suppression may contribute to worsened metabolic outcomes in obesity-related disorders such as MASH.

Unlike BAT, WAT primarily serves as an energy reservoir; however, under certain conditions, WAT can undergo browning, characterized by the expression of *Ucp1* and other thermogenic markers [45,46]. In our study, we did not observe a pronounced difference in IWAT browning between 22 °C and 27 °C, likely because the baseline browning at 22 °C was already low. It is possible that a more robust effect could be observed if mice were exposed to colder temperatures, such as 4 °C, where stronger thermogenic activation would be expected.

In addition to its effects on adiposity, FFD feeding significantly increased *F4/80* gene expression in IWAT, indicating enhanced macrophage infiltration and inflammation. However, temperature had no significant effect on *F4/80* expression, suggesting that diet, rather than thermoneutrality, is the primary driver of inflammation-related changes in IWAT. Increased macrophage infiltration in WAT is a hallmark of obesity-associated inflammation and has been linked to obesity, insulin resistance, and related disorders [47].

Previous studies have explored the relationship between thermoneutral housing and MASLD/MASH. One study reported that 30 °C exacerbates MASLD but did not observe hepatic fibrosis, despite noting simple steatosis and inflammation [48]. This study used Research Diet #D12492, which derives 60% of its calories from fat, differing from the diet used in our experiment. A more recent study examined the impact of thermoneutral housing (29 °C) on the progression of MASLD and concluded that coupling thermoneutral housing with a Western diet for 16 weeks did not lead to significant disease progression in either sex, although molecular analyses indicated priming of immune-related and fibrotic pathways [49]. This study used Research Diet D12079B, identical to the diet in our study, but did not include high-fructose corn syrup. Additionally, their study duration was 16 weeks, compared to our 20-week protocol. When combined with our findings, these results suggest that fructose is a key driver of MASH progression. However, the duration of dietary exposure may also play a critical role, as their study was four weeks shorter than ours. Excessive fructose consumption has been linked to increased systemic inflammation, and studies have shown that fructose promotes leaky gut, endotoxemia, and liver fibrosis through CYP2E1-mediated oxidative and nitrative stress [50].

Although numerous preclinical models have been developed to recapitulate the stages of MASH, few achieve fibrosis while closely mimicking human pathogenesis [51,52]. Our study successfully addressed this gap. Among dietary modification-induced MASH models, high-fat diets have been identified as one of the most effective in replicating human MASH. However, very few studies have investigated how housing temperature affects MASH development. Our findings provide clear evidence that near-thermoneutral temperature significantly alters the trajectory of MASH progression.

Several limitations of this study should be acknowledged. First, histological and plasma lipid analyses were performed on a representative subset of four mice per group because of budget constraints associated with fee-for-service analyses at external core facilities. The selected mice had body weights closest to the group mean and were considered representative of their respective groups; however, we cannot exclude the possibility that the smaller sample size reduced the statistical power of these analyses.

Second, thermogenic function was not directly assessed. Although changes in the expression of key thermogenic markers, including UCP1 and other BAT-associated genes, suggest altered thermogenic capacity, they do not directly reflect tissue-specific or whole-body thermogenic activity. Future studies incorporating direct measurements of energy expenditure, oxygen consumption, BAT temperature, or other functional assessments will be needed to further confirm the effects of housing temperature and dietary intervention on thermogenic function.

Finally, histological evaluations were not performed in a blinded manner and were not accompanied by standardized quantitative assessments, such as NAS-like scoring, fibrosis staging, or digital morphometric analysis of lipid droplet size or collagen-positive areas. Although the histological observations, together with plasma biochemical and molecular findings, support the presence of aggravated MASH-associated pathological changes in mice housed at 27 °C, these findings should be interpreted with caution. Future studies incorporating blinded histological evaluation and quantitative image analysis will further strengthen the assessment of MASH progression.

Beyond these methodological considerations, future studies should explore whether thermoneutrality-induced metabolic alterations can be mitigated through dietary or pharmacological interventions, such as targeting BAT activation and adipose browning. Additionally, investigating the long-term effects of thermoneutrality on MASH progression and liver fibrosis resolution will provide deeper insights into the metabolic mechanisms underlying liver disease.

## 4. Materials and Methods

### 4.1. Animals

Male C57BL/6J mice (9 weeks old) were purchased from Jackson Laboratory (Bar Harbor, ME, USA) and acclimated for one week under standard conditions, including a chow diet, a room temperature of 22 °C, ad libitum access to water, and a 12-h light-dark cycle. After acclimation, the mice were housed at either 22 °C or 27 °C and fed one of two diets: a control chow diet (CHD) (Picolab Rodent Diet 20, LabDiet) or a “fast food diet” (FFD) (D12079B, containing 40% of calories from fat and 0.2% cholesterol, Research Diets) for 20 weeks. The animals’ body weights at the beginning of the experiment are provided in Supplementary Table S1. Mice on the CHD received regular drinking water, while those on the FFD were provided with water supplemented with fructose (23.1 g/L) and glucose (18.9 g/L). All mice had ad libitum access to food and water. Food intake and body weight were monitored weekly.

At the end of the experiment, mice were humanely euthanized using CO_2_. Blood was collected from the portal vein into EDTA-coated tubes. The liver was dissected into individual lobes (left lateral, left medial, right lateral, right medial, and caudate lobes), snap-frozen in liquid nitrogen, and stored at −80 °C. Additional tissues, including cranial thigh muscles, spleen, brain, kidneys, inguinal fat, gonadal fat, retroperitoneal fat, and interscapular BAT, were also collected. Each tissue sample was divided into three parts and stored in RNAlater, formalin, or liquid nitrogen, respectively.

### 4.2. Quantitative Real-Time PCR

Total RNA from liver and adipose tissue was extracted using TRIzol reagent (Thermo Fisher Scientific, Waltham, MA, USA) according to the manufacturer’s instructions. Total RNA abundance was quantified using a NanoDrop ND-1000 spectrophotometer (NanoDrop Technologies, Wilmington, DE, USA). Reverse transcription was carried out using a Maximal First Strand cDNA Synthesis Kit for RT-qPCR with dsDNase (Cat. No. K1671; Thermo Fisher Scientific, Waltham, MA, USA) according to the manufacturer’s instructions. The mRNA expression of target genes and the reference gene, *36b4*, was measured quantitatively using PowerUpTM SYBRTM Green Master Mix (Applied Biosystems, Austin, TX, USA). PCR reactions were run in a 96-well format using an Eppendorf Mastercycler^®^ ep realplex instrument (Eppendorf SE, Hamburg, Germany). Cycle conditions were 50 °C for 2 min, 95 °C for 2 min, and then 40 cycles of 95 °C for 15 s and 60 °C for 1 min. Relative gene expression was calculated using the 2^−ΔΔCT^ method, and the reference gene, *36b4*, was used for normalization. All primer sequences are listed in Supplementary Table S2.

### 4.3. Histology and Immunohistochemistry

Hematoxylin and eosin (H&E) staining and trichrome staining were performed by the Department of Pathology at Texas Tech University Health Sciences Center (TTUHSC). Briefly, liver and adipose tissues were fixed in 10% phosphate-buffered formalin, embedded in paraffin, and cut into 5 μm sections before being processed for H&E staining and trichrome staining. Picrosirius Red staining was performed according to the protocol provided by the manufacturer (Abcam 150681, Abcam, Waltham, MA, USA).

Immunohistochemistry (IHC) staining was performed as previously described [53]. Briefly, formalin-fixed IWAT samples were embedded in paraffin and sectioned at 5 μm by the Department of Pathology at TTUHSC. Deparaffinized and rehydrated sections were incubated overnight at 4 °C with a primary anti-UCP1 antibody, followed by a biotinylated secondary antibody for 1 h. Signal detection was performed using an avidin-conjugated horseradish peroxidase (HRP) system with diaminobenzidine (DAB) as a substrate, following the Vectastain ABC kit manufacturer’s instructions. All histological images were captured using an EVOS^®^ Auto Fluorescence Microscope (Thermo Fisher Scientific, Waltham, MA, USA).

### 4.4. Liver Function and Plasma Lipid 

Plasma alanine aminotransferase (ALT) and aspartate aminotransferase (AST) levels, along with a lipid panel including total cholesterol, triglycerides (TGs), high-density lipoprotein (HDL), low-density lipoprotein (LDL), and very-low-density lipoprotein (VLDL), were measured by the Clinical and Analytical Laboratory at the Nutrition Evaluation Lab, Jean Mayer USDA Human Nutrition Research Center on Aging, Tufts University, following their established protocols.

### 4.5. Sample Selection for Histological and Plasma Lipid Analyses

Eight mice were included in each experimental group. Gene expression analyses were performed using samples from all 8 mice per group. Due to budget limitations associated with fee-for-service analyses at external core facilities, histological analyses and plasma lipid measurements were performed using a representative subset of 4 mice per group. The four mice selected for these analyses had body weights closest to the mean body weight of their respective groups. No animals were excluded from the analyses. Histological evaluation was performed using standardized procedures; however, the evaluator was aware of the experimental group assignments.

### 4.6. Statistical Analysis

All data are presented as mean ± SEM. Statistical analysis was performed using Prism 10.4.1 (GraphPad Software, Inc., San Diego, CA, USA). Two-way analysis of variance (ANOVA) followed by Tukey’s multiple-comparisons test was used to determine the differences among groups. Differences were considered statistically significant at *p* < 0.05. In the figures, different letters indicate statistically significant differences among groups; groups sharing the same letter are not significantly different, whereas groups with different letters differ significantly (*p* < 0.05).

## 5. Conclusions

In conclusion, our study demonstrates that housing at 27 °C, a near-thermoneutral temperature, is associated with exacerbated MASH progression in FFD-fed mice, characterized by increased hepatic lipid accumulation, fibrosis, and inflammation, together with reduced expression of BAT thermogenic markers and adipose browning-related genes. These findings highlight the potential importance of environmental temperature as a factor influencing metabolic disease phenotypes and suggest that alterations in adipose tissue thermogenic capacity may be associated with MASH progression. Future studies involving direct assessment and manipulation of BAT and beige adipocyte function are needed to determine their causal roles in the development of MASH and to explore potential therapeutic strategies targeting thermogenic pathways.

## Data Availability

The original contributions presented in this study are included in the article/Supplementary Materials. Further inquiries can be directed to the corresponding authors.

## Supplementary Materials

The following supporting information can be downloaded at: https://www.mdpi.com/article/10.3390/ijms27177621/s1.
