# Supplementary material for: Housing at 27 °C, a Near-Thermoneutral Temperature, Exacerbates Metabolic Dysfunction-Associated Steatohepatitis in C57BL/6J Mice
Source: Int J Mol Sci. 2026 Aug 25;27(17):7621. doi: 10.3390/ijms27177621 (PMC13566636; doi:10.3390/ijms27177621)
Supplement: Supplementary file 1 [file ijms-27-07621-s001.zip › ijms-4430337-supplementary.pdf]

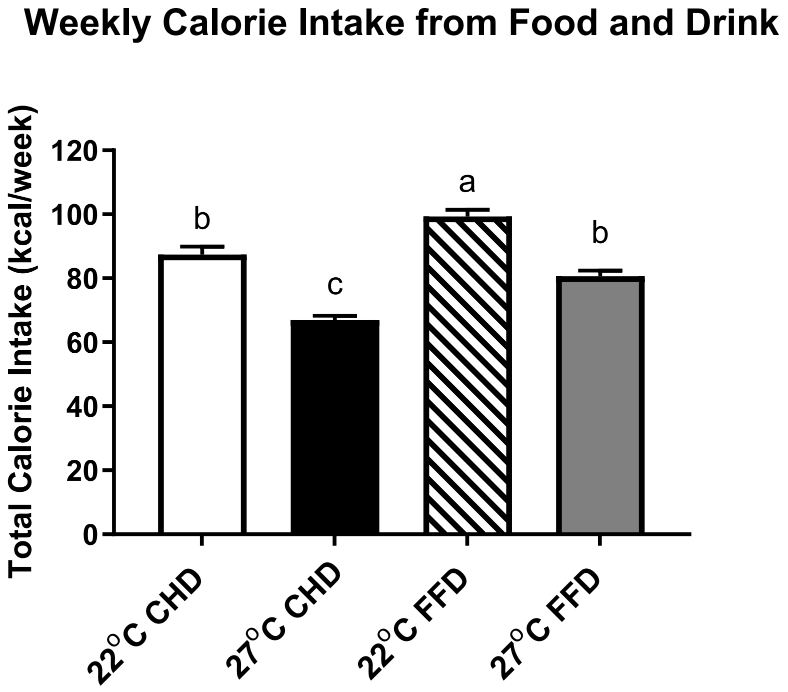

Supplementary Figure S1. Effects of housing temperature on weekly total caloric intake

Weekly food and fluid intake were measured throughout the 20-week dietary and housing intervention. Total caloric intake was calculated as the sum of the energy obtained from food and the supplemented drinking solution. Total caloric intake is presented as kilocalories per week (kcal/week) for chow diet (CHD)-fed mice housed at 22°C, CHD-fed mice housed at 27°C, fructose- and fat-fed diet (FFD)-fed mice housed at 22°C, and FFD-fed mice housed at 27°C. Data are presented as the mean ± SEM. Weekly total caloric intake was analyzed using ordinary two-way ANOVA with housing temperature (22°C vs. 27°C) and diet (CHD vs. FFD) as factors, followed by Tukey multiple comparisons test. Statistical significance was defined as $P < 0.05$. Different letters indicate statistically significant differences.

Supplementary Table S1. Baseline Body Weight by Experimental Group

	22°C CHD	22°C FFD	27°C CHD	27°C FFD
Minimum (g)	22.60	22.70	21.00	22.00
Maximum(g)	25.80	25.90	25.60	25.30
Range (g)	3.200	3.200	4.600	3.300
Mean (g)	24.04	24.36	23.88	24.26
Std. Deviation (g)	1.324	1.140	1.347	1.202
Std. Error of Mean (g)	0.4683	0.4031	0.4761	0.4251

Supplementary Table S2: Primer sequences for C57BL/6J mice

Gene symbol	Primer sequences (5' → 3')	
36b4	Forward	GCTTCGTGTTCACCAAGGAGGA
	Reverse	GTCCTAGACCAGTGTTCTGAGC
Acc1	Forward	ACCGTCTGCTGGGAAGTTAA
	Reverse	CTATCACACAGCCAGGGTCA
Adgre 1	Forward	TGAGTGCACCCAAGATCCAT
	Reverse	TAACCAAGATCCCTGCCCTG
Ccl5	Forward	TGCCAACCCAGAGAAGAAGT
	Reverse	AGATGCCCATTTTCCCAGGA
Cd137	Forward	GCAGCTCAAGAGGAAGATGC
	Reverse	TGTAAGAACAGGGTGGTGGG
Cidea	Forward	AGAAGGTCCTACTGACCCCC
	Reverse	ACCCGGTGTCCATTTCTGTC
Col1a1	Forward	TGGAGATGATGGGGAAGCTG
	Reverse	ATCTCCTTTGGCACCATCCA
Col3a1	Forward	CCCCTGGTTCTTCTGGACAT
	Reverse	TGGGCCTTTGATACCTGGAG
Elovl3	Forward	ACAGAGGCACACACAAACAC
	Reverse	GATAGGGAAGCAGGGTCTCC
F4/80	Forward	TGAGTGCACCCAAGATCCAT
	Reverse	TAACCAAGATCCCTGCCCTG
Fasn	Forward	GAAGGCTGGGCTCTATGGAT

	Reverse	ATGCCTCTGAACCACTCACA
Il1a	Forward	AGCAGCCTTATTTCGGGAGT
	Reverse	ATCATATGTCGGGGTGGCTC
Il6	Forward	CTGGGGATGTCTGTAGCTCA
	Reverse	CTGTGAAGTCTCCTCTCCGG
Il12b	Forward	TTGAAAGGCTGGGTATCGGT
	Reverse	AGCCAGCTCCTCATGAATGT
Leptin	Forward	TGGGGTTTTGGAGCAGTTTG
	Reverse	CTGTCACTCTTTCCCGGTCT
Mcp1	Forward	AGGTGTCCCAAAGAAGCTGT
	Reverse	ACAGAAGTGCTTGAGGTGGT
Nos2	Forward	GTTTGACCAGAGGACCCAGA
	Reverse	GTGAGCTGGTAGGTTCCTGT
Prdm16	Forward	CCGCTGTGATGAGTGTGATG
	Reverse	GGACGATCATGTGTTGCTCC
Srebp1c	Forward	CCCACCTCAAACCTGGATCT
	Reverse	AAGCAGCAAGATGTCCTCCT
Tgfb1	Forward	TCGCTTTGTACAACAGCACC
	Reverse	ACTGCTTCCCGAATGTCTGA
Tmem26	Forward	TGCTGTTCCTGTTGCATTCC
	Reverse	CAGGTACACAAAGATGGCCG
Tnfα	Forward	GACCCCTTTACTCTGACCCC
	Reverse	AGGCTCCAGTGAATTCGGAA
Ucp1	Forward	GCTTTGCCTCACTCAGGATTGG

	Reverse	CCAATGAACACTGCCACACCTC
